# Virtual Classrooms and Their Challenge of Interaction—An Evaluation of Chat Activities and Logs in an Online Course about Digital Medicine with Heterogeneous Participants

**DOI:** 10.3390/ijerph191610184

**Published:** 2022-08-17

**Authors:** Julia Nitsche, Theresa Sophie Busse, Sven Kernebeck, Jan P. Ehlers

**Affiliations:** Department of Didactics and Educational Research in Health Science, Faculty of Health, Witten/Herdecke University, 58448 Witten, Germany

**Keywords:** education, digital medicine, technology enhanced learning, e-interaction, digital competencies, technology, online education, computer literacy

## Abstract

Learning digital competencies can be successful if the information is also tried out immediately using interactive elements. However, interactive teaching poses a particular challenge, especially in large group formats. Various strategies are used to promote interaction, but there is little known about the results. This article shows different strategies and evaluates their influence on the interaction rate in a large group course over two terms that teaches digital medicine. Log files and participation in surveys as well as participation in chat were quantitatively evaluated. In addition, the chat messages themselves were evaluated qualitatively. For the evaluation, relation to the total number of participants was particularly relevant in order to be able to determine an interaction rate in the individual course sessions. A maximum average interaction rate of 90.97% could be determined over the entire term while the participants wrote an average of 3.96 comments during a session in the chat. In summary, this research could show that interactive elements should be well planned and used at regular intervals in order to reap the benefits.

## 1. Introduction

Although the megatrend of digitization affects the healthcare sector and is often discussed in the news and media, it still plays a small role in the academic training of healthcare professionals [1]. Outside of the Coronavirus pandemic, digital media are neither widespread nor an integral part of medical studies [2]. Therefore, it is important to prepare both lecturers and learners in academic training across disciplines for digital teaching in terms of content and methodology [3]. At the same time, digitization in healthcare is a fundamental process of change that is already a practical reality in parts and an element of routine medical work. However, the German National Competence-based Learning Objectives Catalogue for Medicine (NKLM) and the German Masterplan 2020 define measures for the acquisition of a wide range of competencies, but digital competencies are not listed there [4,5]. Nevertheless, the upcoming NKLM 2.0 includes, for example, a learning objective on the fundamentals of machine learning and artificial intelligence systems. Now, several working groups are dealing with its further design.

However, digitization in teaching also plays a crucial role in other departments. Bond et al. (2020) published an overview of educational technologies in use (Zoom, Microsoft Teams, Adobe Connect, etc.) and their impact on student engagement. In the focus are the arts and humanities, education and natural sciences as well as mathematics and statistics [6].

Regarding the need of digital competencies for healthcare professionals, it is crucial to make it visible in lecture methodology and thereby offer the opportunity to use them while learning. The german Witten/Herdecke University (UW/H) has been offering a cross-faculty online course on “Digital Medicine—How will data change the way we treat” since the winter semester 2016/2017 in the spirit of an interdisciplinary approach for students from different faculties (health, business and society).

The significant interest for this course format is reflected in the high number of participants. Starting with about 30 students in the winter semester 2016/2017, up to 300 students (2022: total 2.921 students at UW/H) are currently enrolled in this course. This presents the lecturers with both technical and didactic challenges. The content and the course itself are intended as an interactive format and should always allow students to discuss and express their opinions. It becomes apparent that the degree of digitization and interaction within the course must also be adapted to the specific topic to be able to convey the content in a didactical manner [7].

Even before the Coronavirus pandemic, it would have been expected that digital teaching could have been mastered both technically and didactically. Individual challenges, however, provide for differentiated specifics, so that there is no secret recipe or one path for all. However, the digital transformation of university teaching can also be understood as a long-lasting innovation process that requires change. In this framework, further thoughts must also include changes in structures and processes, which in turn leads to a cultural change with the adaptation of roles and competencies [8,9]. With this understanding, it becomes clear why digital courses pose didactic challenges and why content from classroom teaching cannot simply be presented in a digital space and assumed to be understood in the same way as in classroom teaching. Appropriate technical and didactic preparation of lecturers for a digital educational situation (in large groups) must be implemented [10]. The rethinking and further qualification often also requires an additional effort, which at first seems uncomfortable [11].

However, it becomes apparent that this additional effort is rewarded by a high rate of interaction with the students. Interactive teaching elements in large group formats are considered challenging [12,13]. In the digital learning context, the significant influence of interactive elements on activity levels and learning content assessments is evident [14,15]. It is found that activating participants through regular surveys also promotes more activity and content interaction overall. The importance of activating through queries is confirmed in the literature [16], and queries are recommended as a worthwhile goal [17]. Student engagement and interactivity can also be measured using chat communication [18]. With chat, interactivity during the course can succeed and lead to a better absorption of the learning material, as well as the formation of opinions [19]. The particular relevance of interactivity in online formats is also evident in the meta-analysis of Cook and colleagues [20]: when online learning in the healthcare professions is designed to be interactive, its effectiveness is no different from traditional face-to-face formats.

On the one hand, the course with all its interactive elements described helped prepare participants for digitization in both technical and practical terms. On the other hand, they also provided educational work to train, expand and question their own attitudes and acceptance of digitization in healthcare. This competence is crucial in order to not only bring along a basic understanding of digitization in the (later) professional field, but also to be able to critically question the consequences of digitization for one’s own professional field and to know the opportunities and risks.

In order to be able to convey this content, the course “Digital Medicine—How will data change the way we treat” focuses on the greatest possible interaction with participants and also between them. The aim of this study is to review these interactive elements in terms of their success and to show the results in order to close the research gap. The research group poses the following research questions:

- Can different forms of (meaningful) interaction take place in an online course of this size?
- What interaction rate can be achieved in an online course through simple and continuous interaction elements?

## 2. Materials and Methods

Ethical approval was obtained from the Ethics Committee of UW/H (approval code: 46/2020).

### 2.1. Intervention

To answer the research questions, an interaction rate was determined in the course. The course was held via the virtual meeting software Adobe Connect (Version 11.2) in the winter term 2019/2020 (WT 19/20) and summer term 2020 (ST 20) at the UW/H. Adobe Connect offers several built-in features to manage digital teaching in large groups: presentation modes for different media (image, audio, video and presentation), anonymous and public chat function and (anonymous) polling function, as well as group rooms. In addition, it is possible to download differentiated reports on individual sessions afterwards, showing, e.g., log data and activities. The focus is not only on the thematic exchange about digitization, but also on the application of digitization in particular through interactive elements, combining teaching and method. An acceptance analysis has already been conducted in this course, which shows that the students were satisfied with both course format and course content in previous semesters [21]. Other research also shows that similar courses are worthwhile, as students found the content interesting and the course worth attending [22].

The course itself has had a scope of 2 Credits in the European Credit Transfer and Accumulation System (ECTS) for participation since the WT 2018/2019 and took place weekly on Thursdays from 18–19:30 o’clock. Attendance as a basis for awarding ECTS was verified by login. Participants were asked to log in with their explicit real names in order to be able to assign them to the course registrations. If several participants logged in together with one account, they were required to briefly prove via video at the beginning of the course that they were participating together. During the course, participants did not have the opportunity to share their video or audio themselves. It could be shared after approval by the facilitators. The lecturers, however, had activated their video. Communication occurs therefore mainly via chat.

The course was co-taught with a total of four people as facilitators (J.N., J.P.E., T.S.B., S.K.) responsible for the course. One (varying) facilitator moderated the session and was in contact with the respective guest-lecturer. The other persons provided support by answering the chat, reading out content from the chat and assisting with technical difficulties on the part of the lecturer or the participants, as well as activating the polls. Both the facilitators and external lecturers designed the course content itself. The surveys were also explicitly prepared in order to focus on the content in the course itself. A table (Table 1) with the exact course content can be found below. Particularly relevant are current practical examples to make digitization in healthcare tangible for the participants. Guest lecturers offer the opportunity to convey this.

### 2.2. Participants

Students from the Faculty of Health (Medicine, Dentistry, Psychology and Nursing Science) and the Faculty of Business and Society (Management, Philosophy, Politics and Economics) could participate [21].

The individual topics were presented weekly by lecturers; these were usually invited guests who present their expertise in their field of application, but they could also be students who report on practical use cases that they present with scientific support. Some of the courses were taught by the facilitators themselves. The course lived primarily through the interaction between participants and lecturers/facilitators. In order to create as much interaction as possible in an online course with up to 280 simultaneously registered participants, the following measures were used: The chat itself served as a direct means of contact with the participants. Particular attention has been paid to ensure that the participants quickly make themselves known through short response options and that a sense of community could develop (everyone else responds as well). Answer options for questions to be answered in the chat were sometimes defined with 1 and 0 in a dichotomous answer format of a question. Questions were asked via chat to play them back aloud before they were answered. One or two co-facilitators kept an eye on the chat and answered questions that had already been asked or put them on hold for later and informed the participants accordingly. Participants usually wrote the messages in the “open to all” mode, which means that all participants could see the written message. In addition, there was also the function to send private messages to individual persons. If private messages were written to the (co-)facilitators, they were also answered privately in the chat. If the question and answer were relevant to everyone, the question was repeated anonymously for everyone and then answered verbally.

### 2.3. Data Collection

Through a targeted use of the query function and integration of the participants in the course of the event by actively addressing and verbally repeating the questions and opinions on the presented content through the lecture, involvement of the participants should be increased.

Both internal and external lecturers held the dates of the course. Nevertheless, the internal organizers always pointed out the special circumstances and possibilities for activation to external experts before the event. The activation measures were used regularly. The number and extent of these measures depended heavily on the lecturers. In addition to the activities in the chat, participants could take part in surveys. These were used to gain a picture of the understanding generated among stakeholders. The results could be presented directly and were also available afterwards.

### 2.4. Data Analysis

To be able to evaluate the interaction of the participants among each other and with the lecturers, the number of participants and comments, number/percentage of commenting participants and participation in polls were evaluated descriptively for all dates of the courses and the chat messages were analyzed.

Therefore, log data (list of participants, chat logs and survey logs) were downloaded. Using Microsoft Excel (Version 2016), the list of participants and survey logs as well as chat logs were presented for quantitative evaluation. In addition to the pure activity, the content of the messages was also crucial to enable an assessment in terms of the quality of the interaction and its impact. The classified chat messages were evaluated descriptively. Qualitative content analysis oriented by Kuckartz [23] (using MAXQDA 2020) with an inductive approach was used for the chat logs.

## 3. Results

A large number of participants in both terms attended the course. In the following, the differences between terms as well as between individual course dates are presented. Here, the numbers of participants and the activity in the chat as well as the contents of the chat interaction are considered.

**Figure 1:**
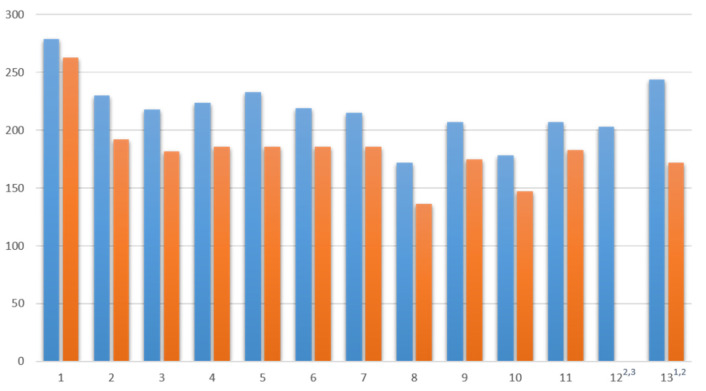
All participants (blue, left) and active participants (orange, right) for the course in WT 19/20. ^1^ Elective date, ^2^ Additional days due to the length of the semester, ^3^ Log files for this date are not available due to technical problems.

**Figure 2:**
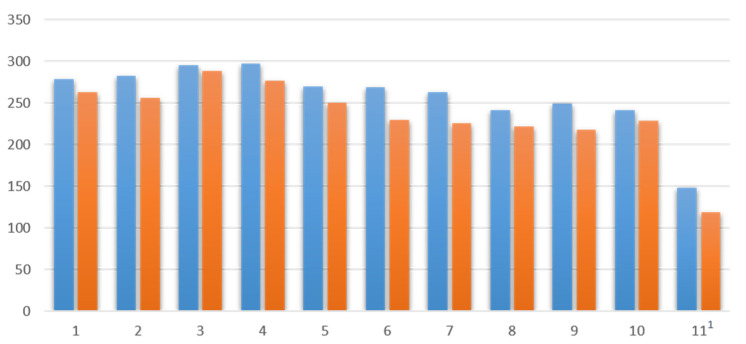
All participants (blue, left) and active participants (orange, right) for the course in ST 20. ^1^ Elective date.

First, participants were evaluated in terms of their general activity during the individual events. Figure 1 and Figure 2 show a comparison of all participants and active participants (using the chat at least once) in each session measured by messages in the chat.

In the WT 19/20, the mean value of participants across all sessions was 213.15 (SD 20.4) and that of active participants was 175.9 (SD 17.1). Comparatively, the mean value of participants across all sessions in the ST 20 was 257.73 (SD 41.2), while the mean value of active participants was 234.45 (SD 44.9). Both the number of participants and the number of active participants increased from the WT to the ST. A look at the rate of active participants shows that this increased, too: 82.5% (WT 19/20) vs. 90.97% (ST 20).

The analysis of the messages in the chat itself provides information about how many messages were sent per participant on average and what was the content orientation of these messages. In the WT 19/20, the mean value of comments in each session was 493.8 (SD 120.3). In terms of active participants, the mean value of comments per participant was 2.8. In ST 20, a mean value of 922.45 (SD 418.9) comments were sent in each session. In terms of active participants, each participant sent a mean value of 3.94 comments. This also shows an increase of 1.14 messages more per active participant from WT 19/20 to ST 20.

Within the qualitative content analysis, three categories of interaction emerged (see Table 2).

Table 3 shows a comparison of the individual chat categories of the two semesters under consideration.

On average, 63.2% of all comments over the entire WT 19/20 could be categorized into “social interaction”, whereas, 13% of the comments can be classified in the “technical interaction” category and 23.8% of the comments fall into the “interaction on topic” category. A slightly shifted distribution can be found in the ST 20: 40.77% of comments were social in nature, while 5.71% of comments were related to technology. The “on topic” comments increased to 53.69%. Accordingly, not only did the number of participants and activity increase, as shown earlier in the course, but the types of comments made were also steered away from the “social” category toward the “on topic” category.

While polls were conducted in 12 of 13 sessions in the WT 19/20, this method was only used in 7 of 11 sessions in the ST 20. Here, the participation range spans 18.9–92.9% across the surveys in the WT 19/20 and 57.74–92.58% in the ST 20. Again, this indicates an increase in activity from the winter to the summer semester. Regardless, the mean value of participation in polls (built-in function) across all dates in the WT 19/20 was 74% (SD 14.1), while in the ST 20 this was 80.54% (SD 9.85).

## 4. Discussion

The online format used in this study enabled the effective teaching method of an interactive learning session in large groups with frequent questions to participants and open discussion sessions. The results show an example in which interactions among and with participants in large groups can be used in the online teaching format. Using information and communication technologies, the active role shifts from the lecturers to the participants, who can interact with the lecturers as well as with each other [24]. It can be assumed that the increase in content-related comments in the ST 20 compared to the WT 19/20 is not a random product, but a targeted use of the inquiry function and reference to the students during the lectures by actively addressing and verbally repeating the questions and opinions on the presented content by the lecturer. In ST 20, even more attention was paid to embedding the polls in the content and verbalizing both the question itself and the subsequent results. The lecturers repeated questions asked in the chat verbally before they were answered. In some cases, participants were also addressed directly to clarify queries.

In another study, the participation and interactivity of students in e-learning pathology courses was also measured by means of communication in the chat [18]. It was found that the maximum participation rate in the first session (76.26%) was 257 students in total, and this rate decreased over the course of the semester. Even if such a pattern cannot be clearly shown in our results, it is evident that there is an increased activity rate especially in the first third of the semester. Particularly at the beginning of both semesters, it becomes apparent that there is an increased social and technical exchange in the chat. This form of exchange also makes a valuable contribution to creating a sense of community in a digital space and to opening up a room of trust. It enables participants to express their opinions and ask questions as the course progresses, even if they do not know the majority of the participants personally due to the composition of the group. This might also be relevant for online teaching under pandemic conditions (e.g., through isolation). Regarding this, it is interesting to mention a study that shows that both instructors and students report positively about interactive learning that also creates a sense of community [25].

Moreover, this interactivity is also demanded by students in online courses, both with each other and with faculty. If it takes place, they benefit from it [26].

The administrative organization of the course requires at least two responsible persons per term due to the large group in the described format. The above-described form of division and use of co-teaching may be a solution to the practical difficulties of a synchronous interaction activity by the lecturer, the consequence of which is that few studies have investigated this [27].

An interactive digital course can also contribute to the training of students for their future careers. The increasing digitization in healthcare brings with it, for example, video consultation hours [28]. Here, an onlinecourse with various interactive elements can promote informal learning of such formats. However, students are also well educated through such a format in the context of digital exchanges with other healthcare professionals and learn informal behaviors and interactive manners. Even in the case of online continuing education, as is to be expected increasingly in medicine in the future [29], the students are prepared by such a course and can thereby also use an expanded circle of continuing education.

Furthermore, it is to be discussed how to compare the results with face-to-face teaching with such a large group. Is implementation in this format with such participation even possible? Probably only if audience response systems or other educational technologies that enable interactivity [30] are used. Audience response systems, for example, are a simple tool to create an interactive environment [31]. At this point, a stable and well-developed technological infrastructure is conducive to more efficient use [32]. In addition, transparent handling of results is necessary to allow participants themselves to gain insight into the opinions of others. If this is observed, both students and lecturers are enthusiastic and believe that learning is improved [33]. There is no provision for bilateral exchange of ideas with each other as part of the continuing face-to-face teaching, but it would provide an opportunity for others to participate if they are not directly involved in the exchange. An additional chat room could be set up here, which the participants then visit virtually while taking part in the face-to-face teaching. A similar combination has been tried in the past in another study and was found to be positive [34]. However, it would then additionally require a moderator as well as technical equipment on both sides and a form of transparency for the lecturers. Future research is needed here on the difference of whether the event is held in person or online.

From the perspective of self-criticism, we acknowledge that we did not sufficiently consider the specific content of each session. The analyses might have been more in-depth if dynamics in the chat progressions and conversations could have been illuminated. Additionally, more detailed results could have been obtained if we had also meticulously tracked interactivity patterns related to the content and then analyzed them coherently.

## 5. Conclusions

A challenge in digital interactive teaching in large groups requires commitment from the lecturers. Not only does the content have to be adequately prepared, but the interaction itself also has to be prepared and carried out in a complex manner. The most important thing here is to engage in discourse with the participants at regular intervals by means of queries and questions. The anonymity of such large group online formats can also be an advantage, provided it is consciously accepted. In the world of increasing digitization, a trend also observed in healthcare, an interactive course with such a thematic focus can also be helpful for the students in their future careers (e.g., video consultation or digital training). Finally, it also includes empowering the digital health literacy of patients, who are all participants.

It is essential to reinforce these advantages because increasing group sizes can actually promote reticence on the part of participants, especially with regard to the free expression of opinions and the asking of comprehension questions [35].

## Figures and Tables

**Table 1:** Program overview for Digital Medicine in WT 19/20 and ST 20.

Session	Topic	
	Winter Term 2019/2020	Summer Term 2020
1	Introduction Digital Medicine	Introduction Digital Medicine
2	Artificial Intelligence	COVID-19
3	Project presentation XPOMET	Care robotics
4	Homo digitus	E-learning
5	Digital insulin pump and Ivena	Online psychotherapy
6	Social Media and learning with webinar	Apps in medical sector
7	Project presentation Chatbots@FOAMed	Digital processes in medicine
8	Electronic medical records	Homo digitus
9	Project presentation da Vinci	Virtual reality in medicine
10	Project presentation Biotronik	Digitization in pharmacy
11	eHealth Literacy	Site Visit ^1^
12 ^2,3^	Data protection in medical practice	
13 ^2^	FutureMedTalk ^1^	

^1^ Elective date, ^2^ Additional days due to the length of the semester, ^3^ Log files for this date are not available due to technical problems.

**Table 2:** Categories of chat messages with sample quotes.

Categories	Description	Sample Quotes
Social interaction	This category includes statements that promote a purely social exchange, whether among the participants or even with the presenters, but make no substantive contribution to the topic under discussion.	“Good Evening”, “Sorry that I’m late”
Technical interaction	This category includes statements dealing with technical problems or suggestions related to the functioning of the platform itself. It does not include statements that revolve around technical content in the session.	“I have problems with the internet connection”
Interaction on topic	This category includes statements that relate to the topic of the session, either as questions or as comments. It does not include statements that are, for example, thanks to the presenter (social interaction).	“A nurse could make house calls with a digitally connected doctor”

**Table 3:** Qualitative evaluation of chat messages in WT 19/20 and ST 20.

	Winter Term 2019/2020			Summer Term 2020		
Date	Social Interaction (%)	Technical Interaction (%)	Interaction on Topic (%)	Social Interaction (%)	Technical Interaction (%)	Interaction on Topic (%)
1	64.30	17.62	18.54	36.54	18.63	44.93
2	64.05	14.15	23.71	19.37	8.27	72.36
3	54.64	10.93	34.77	28.39	3.24	68.18
4	79.90	16.95	3.15	30.55	3.56	66.21
5	75.84	15.34	8.40	53.79	5.40	40.80
6	57.34	9.44	33.39	57.38	1.82	40.98
7	58.58	15.12	26.16	92.86	5.36	4.02
8	82.15	10.46	7.38	47.83	4.23	48.16
9	58.86	20.23	20.90	64.34	3.49	32.17
10	84.64	9.34	6.02	36.88	2.81	60.42
11	45.28	8.92	45.98	55.13 ^1^	4.40 ^1^	40.47 ^1^
12 ^2^	n.n ^3^	n.n. ^3^	n.n. ^3^			
13 ^2^	68.96 ^1^	7.42 ^1^	23.63 ^1^			
**Ø of all dates**	63.2	13	23.8	40.77	5.71	53.69

^1^ Elective date, ^2^ Additional days due to the length of the semester, ^3^ Log files for this date are not available due to technical problems.

## Data Availability

The corresponding datasets of this study are available from the corresponding author upon reasonable request.
